# Assessing spinosad effect on honey bee olfactory conditioning using a microcontroller-based device

**DOI:** 10.3389/finsc.2026.1785989

**Published:** 2026-05-14

**Authors:** Gabriele Rondoni, Francesca Napoli, Elena Chierici, Eric Conti

**Affiliations:** Department of Agricultural, Food and Environmental Sciences, University of Perugia, Perugia, Italy

**Keywords:** *Apis mellifera ligustica*, associative learning, insect behaviour, non-target effect, odour

## Abstract

Honey bee health is adversely affected by numerous biotic and abiotic stressors, such as the extensive use of pesticides, which reduce survival and, even at sublethal doses, impair fitness and behaviour, including learning ability. The proboscis extension reflex (PER) is a widely used method to investigate associative learning in different insect species, such as *Apis mellifera ligustica*, when exposed to biotic and abiotic stresses. PER protocols require careful insect handling and accurate timing of stimulus application for effective conditioning and precise learning behaviour assessment. Despite the considerable amount of literature on this topic, information on automatising PER protocols for evaluating a large number of insects is limited. In this study, under laboratory conditions we evaluated whether exposure to different lethal concentrations (LC_0.2_ and LC_20_) of the natural bioinsecticide spinosad affects olfactory learning in groups of twenty worker bees within the same experimental cycle. For this purpose, we developed and validated an automated, microcontroller-based open-source device designed to standardise key steps of the PER conditioning protocol. Spinosad is a neurotoxic molecule that is known to induce mortality in honey bees, as well as behavioural impairment on locomotion and orientation at low doses. Surprisingly, possible effects on associative learning evaluated through PER are unknown. The sequential steps of the PER protocol were mechanised using a rotating carousel, where the conditioned stimulus was automatically released. The operator manually applied the unconditioned stimulus to the antennae, provided the rewarding solution, and recorded whether each insect exhibited PER behaviour. Bee responses were instantly transmitted to a laptop using a basic serial data transfer protocol and revealed decreased PER response (about 25%) for both spinosad concentrations at 20 min after the last conditioning trial. After small adaptations and validation, we expect that the device can be adopted both in laboratory and field conditions to investigate PER response in other bees, e.g., wild pollinators and bumblebees. This work aligns with current attempts to develop novel tools for monitoring biopesticide effects on pollinator health in changing environments.

## 1 Introduction

Agricultural practices could alter the optimal foraging behaviour of beneficial insects, including pollinators (1–3). Empirical evidence attributes this phenomenon to the effect of stressors, including climate change, fragmentation and degradation of habitats, and pollution by xenobiotics (4, 5). Direct and indirect exposure to pesticides is recognised as a major threat (1, 6–8). Among the different effects that stressors could cause to beneficial insects, sublethal effects are well known (9–15). Common interferences with insect behaviour are disturbances in mate recognition, food foraging, orientation, guarding behaviour, and learning (11, 16–22).

In honey bees, there are established protocols for studying learning ability (23–25). Variation in associative learning can be assessed by observing walking and arresting behaviour, for example, in open arena or T-maze bioassays, or by observing proboscis extension reflex (PER) (26–30). PER is a reflexive behaviour that occurs in response to stimulation of different body parts, such as antennae or tarsi, when they contact sucrose or pollen (31, 32). In classical conditioning, an association is created between a conditioned stimulus (CS) and an unconditioned stimulus (US) (33). For example, in an olfactory PER conditioning protocol, an odour is provided to the honey bee (as CS) together with stimulation of the antennae using a sugar solution (as US). A sugar solution is then offered to the honey bee's ligule to reinforce the CS-US association. After conditioning, it is expected that the honey bee extends its proboscis when the conditioned stimulus is provided alone (23, 31, 34–36). Individuals in good conditions should easily learn to associate the odour with the reward, while those subjected to stress can have impaired learning abilities (11, 37–39).

Considering the complexity of PER protocols, which is mainly due to the need to guarantee the exact duration of sequential steps, automation of the procedure would be extremely beneficial. Some automated systems reported in the literature cover only specific parts of the PER protocol (40–45), whereas others do not envisage the screening of several bees (from one honey bee to maximum twelve) within the same experimental cycle (46–48). Furthermore, the use of open-source devices would make the design and evaluation of learning even more easily accessible and customisable for ecologists and entomologists, as elsewhere demonstrated (29, 49–53). In particular, the use of sensors and a crystal oscillator as a clock standardises the temporal structure of the conditioning protocol across individuals, provides intra-cycle replications, reduces variability induced by the operator, thus ensuring consistent inter-stimulus intervals throughout the experiment.

Here, we assessed under laboratory conditions whether artificial exposure to low concentrations of a natural insecticide (active ingredient: spinosad), namely LC_0_ (control), LC_0.2_, and LC_20_, affects learning and memory retention in *Apis mellifera ligustica* (Spinola) workers. Specifically, we investigated if the lethal concentrations affect the honey bee behavioural performances in olfactory PER conditioning assays. To address this, we designed and evaluated an open-source electromechanical device based on microcontrollers that enables a more standardised application of the experimental procedure. Spinosad acts as an allosteric modulator of nicotinic acetylcholine receptors and a GABA agonist, producing neuroexcitation and paralysis (54). In *A. mellifera*, through contact and ingestion it causes both lethal effects and sublethal effects, namely behavioural impairments of locomotion and orientation, and disruption of sensory processing (55, 56). Surprisingly, the potential impact of spinosad on associative learning, evaluated through PER, remains unknown. We hypothesised an impairment of the PER response in honey bees treated with spinosad.

## 2 Materials and methods

### 2.1 Evaluation of dose-response mortality to the spinosad-based bioinsecticide

Honey bees were sampled during summer 2021 from one hive located in the botanical garden of the University of Perugia (central Italy). Treatment with amitraz (commercial product “Apivar”, Laboratoire Biové, France) against *Varroa destructor* Anderson and Trueman (Mesostigmata: Varroidea) was applied during August, when bioassays were not performed.

The exposure of the honey bee to field residues of the insecticide was simulated by allowing the insect to walk on a previously contaminated surface. The surface was represented by the inner area of a Falcon tube (50 mL volume). The contamination of the Falcon tube was conducted according to the protocol reported in IRAC Susceptibility Test Method, No 30, v. 1.2 (https://irac-online.org/methods/). The protocol describes the procedure for the conversion of the field dose (1.5 L/ha) to ml/cm^2^ of the test tube.

Serial dilutions of Success™ (active substance spinosad 11.6% corresponding to 120 g/l, Corteva Agriscience Italia srl) were prepared from 100% of the field dose. The initial solution was prepared using a water: acetone mixture, which is characterised by high volatility (57). In detail, the insecticide was dissolved in water (750 µL) first, then acetone was added to the solution to reach the final desired volume per Falcon tube (20 mL). The 100% solution (field dose) corresponded to 13.19 µl of Success™ per tube. Serial dilutions of 50%, 25%, 12.5%, 6.25%, 3.13%, 1.56%, 0.78%, 0.39%, 0.20%, 0.10% were made from 100%. Negative control consisted of a water: acetone solution in the v/v ratio as reported above. Each dilution was used to prepare 4 Falcon tubes and 4 nets, used for non-hermetic sealing of the tubes. These were initially placed with a small degree of inclination that gradually decreased from +10° to 0° and gently rotated (automatically at 2 rpm) under a fume hood for several hours until the solvent had completely evaporated (similar to 58). The nets, which provided an additional contact surface for honey bees, were also dipped in insecticide dilutions and dried under a fume hood.

Groups of three bees were transferred to each treated tube for 12 h (daily at 8:00 pm) (similar to 11, 58). Honey bee food (Candifruit, Adea Srl, Italy) was provided as nourishment with a plastic strip (5 mm × 10 mm). At the end of the period, honey bees were transferred to clean tubes (food provided), and mortality was recorded after 24 h. Data were analysed by means of dose-response curve using the *drm()* function from the *drc* package in R, (59). The sublethal concentrations causing mortality levels of 0.2 and 20% were estimated from the model using *ED()* function and were used for subsequent behavioural assays.

### 2.2 PER bioassay

Protocols for insect handling, exposure time, conditioned and unconditioned stimuli, loop duration and number, and memory retention testing were adopted from the literature (1, 11, 60). For the experiment, groups of 25–30 worker honey bees were collected directly from the hive’s landing board in the late afternoon (at 6:00 p.m.). Honey bees were brought to the laboratory and maintained in yellow “Nicot” cages (80 mm × 35 mm × 13 mm, six bees per cage; 25 ± 1 °C, 16: 8 L:D, 65% RH). About 1 g of honey bee food (Candifruit, Adea Srl, Italy) was provided in each cage as nourishment. After approximately 2 h (at 8:00 pm), insects were transferred to a 50 mL Falcon tube (six bees per tube), previously treated with Success™, an insecticide permitted in organic farming and based on spinosad (11.6%, corresponding to 120 g/l; Corteva Agriscience Italia srl). Different insecticide dilutions in water:acetone, causing mortality rates of 0.2 and 20%, respectively, were tested. The specific concentrations were estimated from a dose-response curve obtained as indicated in section 2.1. Honey bees kept in Falcon tube treated with only water:acetone solution served as control group (LC_0_) (see section 2.1 for details). The insects were then transferred to a dark environment and maintained at 25 ± 1 °C and 50 ± 10% RH. After 12 h of exposure to spinosad or water:acetone treated Falcon tube, the honey bees were returned to the “Nicot” cage for 24 h (1 d). Twenty insects were then anaesthetised using CO_2_ to facilitate their handling. Previous studies revealed that quick exposure to CO_2_ did not alter learning ability (61). Each honey bee was inserted into a plastic tube (described in Section 2.3 “Design and evaluation of PER device”) up to its neck. A small piece of cotton wool, fitted inside the tube, provided support to the bee abdomen. The distal part of the tube was crossed by two metal pins that were placed on both sides of the honey bee neck, as a yoke (62). This prevented the insect from escaping the tube while still allowing it to freely move its mouthparts and antennae (62). Honey bee antennae were gently touched with a bristle embedded with 50% sucrose solution to verify individual ability to extend the ligule when properly stimulated. This ensured that only insects displaying a positive PER response were selected for downstream PER conditioning trials (63, 64). Then, each of these twenty restrained bees was positioned in the black plastic cage of the device (see Section 2.3) and left food-deprived for 2 h for acclimatisation. A period of 1–3 h of food deprivation is considered enough to standardise motivation levels (similar to 31, 63). To allow familiarisation with the mechanical air flow, prior to the PER conditioning trials, each bee was subjected to a clean air flow (2.2 L/min) for 15 s and then to a rest interval of 10 min (65). At this step it is expected that honey bees do not extend their proboscis as a merely consequence of the airflow stimulation.

For the olfactory PER conditioning trials, an airflow (2.2 L/min) of the conditioned stimulus (CS; 10 μL of pure linalool; Sigma Aldrich, Italy) was conveyed for 6 s. After the first 3 s, one randomly selected antenna was gently touched with a bristle embedded with 50% sucrose solution (unconditioned stimulus, US). If the insect responded by extending the proboscis, it received the sucrose solution directly on the glossa as a reward. Every conditioning trial was followed by a 10-min break before a new trial was conducted. Five olfactory PER conditioning trials were conducted for the two dose treatments and the control. Memory retention tests were conducted 20 min after the last conditioning trial. During the memory retention tests, honey bees received only the conditioned stimulus, i.e. 6 s linalool (2.2 L/min), the antennae being not exposed to any unconditioned stimulus. If a bee extended its proboscis, a sucrose reward was provided, and this behaviour was interpreted as an indication of successful associative learning. For the experiment, 60 honey bees per treatment were tested. Insects that did not respond to at least one conditioning trial were eventually discarded from the analysis. The temperature in the bioassay room was maintained at 25 ± 1 °C (66).

The PER responses of honey bees during the memory retention test at 20 min after conditioning were analysed using binomial generalized linear models (GLMs). Multiple comparisons procedure was then conducted to evaluate the effect of insecticide concentration (package *emmeans*^67^).

### 2.3 Design and evaluation of PER device

**Figure 1:**
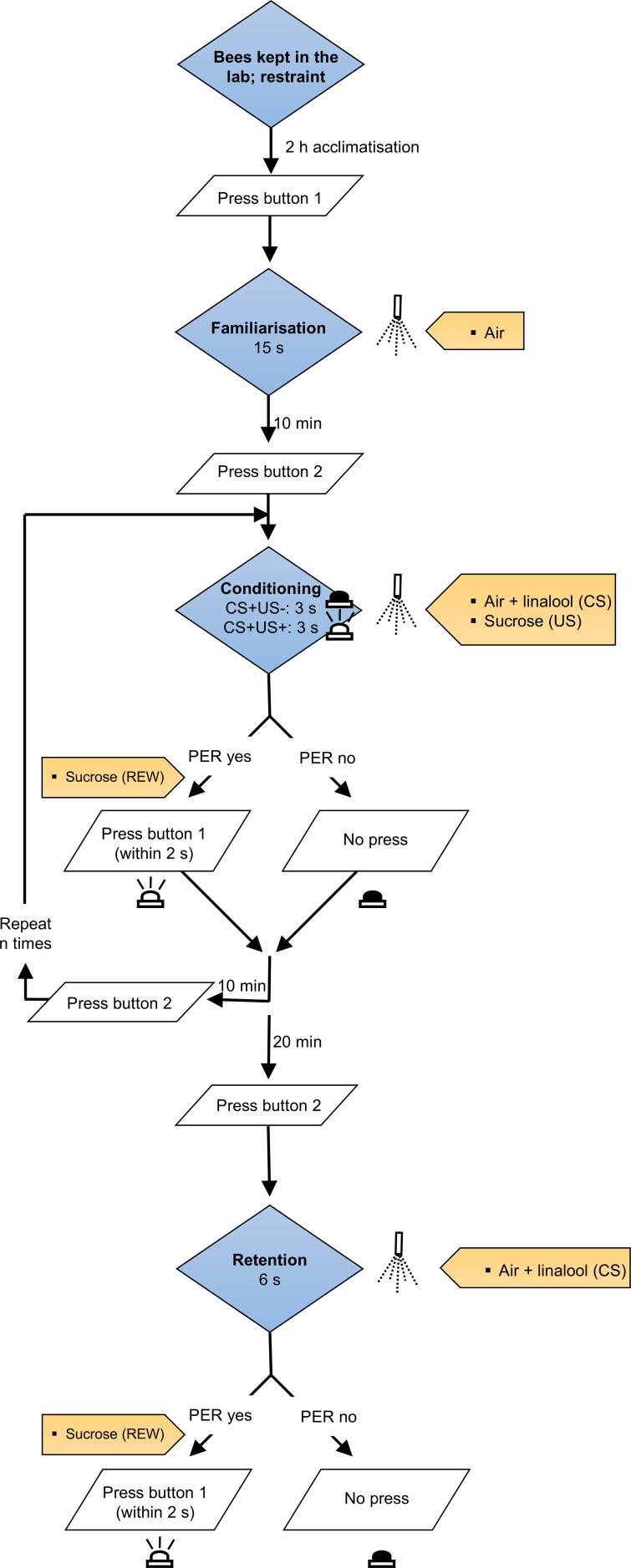
Flow diagram of the procedure implemented to evaluate PER behaviour. “CS”, conditioned stimulus; “US”, unconditioned stimulus; “REW”, reward.

The device consisted mainly of a plastic turntable (50 cm diameter) with 20 black plastic cages (90 mm height × 57.5 mm length × 53.5 mm width each) vertically attached equidistantly to the external side of the turntable (**Supplementary Figure S1**). A unipolar stepper motor (0.8 A, 1.8 degrees/step, Vexta PK245-2B, Oriental Motor Co., Ltd, Japan) was directly connected to the centre of the turntable, allowing its rotation of 18 degrees (corresponding to ten motor steps) clockwise whenever requested. The upper side of each box was centrally perforated (2 cm diameter). A plastic cylinder (2 cm height and diameter) was centrally fixed on the internal lower side of each box. A vertical hole (7 mm diameter, 10 mm depth) was drilled at the top of the cylinder. Before the bioassay, an individual honey bee was restrained at the top end of a plastic tube (7 mm diameter × 50 mm length; for details see 2.1 Insect source and handling), which was then vertically inserted into the cylinder hole. The upper end of the tube was shaped by cutting a piece of the border (4 mm × 4 mm) to accommodate the front part of the insect head. After insertion of the tube into the plastic cylinder, the distance of the bee head from the upper side of the cage was ~3 cm. An air tank provided medical grade compressed air (N_2_:O_2_, 80:20). A pressure gauze and a solenoid valve (12V) controlled air passage at 5 atm. The airflow was then split into two routes, each consisting of a flowmeter and a Dreschel bottle, filled with distilled water for air humidification. One flowmeter regulated airflow at 2.0 L/min, the second flowmeter at 0.2 L/min. Silicone tubes (6 mm internal diameter) were used to connect all parts of the device. The tubes coming from the Dreschel bottles were connected to the two arms (each of 5 cm length, diagonally inserted and forming a 45-degree angle) of a glass Y-shaped connector, which eventually reunified the airflow. A small piece of filter paper (1 × 1 cm) was carefully folded along one side and inserted into the connector arm that receives the lower airflow. At the end of the bioassays, the glass parts were washed with laboratory detergent, rinsed with tap water, then washed with acetone under a fume hood, and heated at 110 °C for 5 h. The plastic tubes sustaining honey bees were washed with laboratory detergent, rinsed with tap water, and conditioned with a nitrogen stream for 15 min. In the familiarisation phase with the mechanical air flow (**Figure 1**), the filter paper was kept clean. Subsequently, the filter paper was removed from the Y-shaped connector arm using a pair of tweezers and soaked with 10 µL of linalool (Sigma Aldrich, Italy) as a conditioned stimulus. The filter paper was dried for 5 min and reinserted into the connector arm. The common arm of the Y-shaped connector conveyed the airflow to a glass tube, bent to form a 90-degree angle (tube length: 20 cm the longest arm, 5 cm the shortest). The shortest end of the tube was vertically positioned above the top of a black box containing the restrained bee. The glass tube was affixed, at its base, to the arm of a high-torque servo motor, which allowed up-and-down movement of the tube. In its lower position, the shortest end of the tube entered the black box to convey airflow at ~2 cm from the honey bee. Subsequently, the tube moved to its upper position, allowing the turntable to rotate. The use of a servo motor ensured the fast repeatability of up-and-down movements of the tube. The turntable was illuminated from above by two 32 W white neon tubes, enveloped in a metal grid cage, to prevent possible noise from electronic devices (68), and was surrounded by black cardboard to minimise visual input from the room (69). The flow rate that exited from the 90-degree bent glass tube was measured with a digital flowmeter (mod. GFM17, Aalborg, USA) to confirm the rapid delivery of the desired airflow (2.2 L/min) to the honey bee whenever planned by the protocol.

Concerning the electronic parts of the device, a stepper motor was connected to the motor driver (L293D, Texas Instrument Inc., Texas) and to a microcontroller (ATmega 328p, Atmel Corp., California) embedded in a preassembled Arduino Uno R3 board for motor direction, steps, and speed control. The crystal oscillator on the board offered accurate time information to the microcontroller. Additionally, the board regulated the movement of the servo motor and controlled a normally open 5VDC, 10 A, 220 VAC relay (Arceli, India). The relay controlled the activation of a 220V/12V AC/DC voltage regulator (Mean Well, Taiwan). When the relay was activated, it allowed power supply to the voltage regulator, which eventually excited the 12V solenoid valve (Univer, Italy), allowing air to flow into the tubing. A 220/5V AC/DC voltage regulator (Mean Well, Taiwan) provided power supply to the board, the motor driver L293D (Texas Instruments Incorporated, Texas), the two motors, and the relay. A 16A magneto-thermic circuit breaker (ABB, Italy) was included for circuit protection. Two buttons (B1 and B2, **Supplementary Figure S2**) were connected to different inputs on the board and allowed the device to fully function. By pressing B1, the system began the familiarisation phase for the 20 restrained insects in sequence (**Figure 1**). After that, pressing B2 started the first loop of the olfactory PER conditioning for each insect in sequence. During the trial loop, after 6 s (3 s CS plus 3 s CS+US), pressing B1 within 2 s was used to indicate that the insect exhibited PER, while no pressure meant no PER response. A light-emitting diode (LED) was included in the circuit. The LED blinked in two occasions, at first to indicate the timing when the operator should provide the unconditioned stimulus (i.e., 3 s after the beginning of the conditioning), and later to eventually confirm the correct pressure of B1 in case of PER response. At the end of the loop, subsequently pressing B2 enabled the device to start a new olfactory PER conditioning loop. Although the unconditioned stimulus (sucrose solution) was delivered manually and PER responses were recorded manually, the device automated the sequential presentation of the conditioned stimulus, airflow delivery, inter-trial intervals, and rotation among individuals. This configuration allowed the standardised conditioning of up to 20 bees within a single experimental cycle, minimising timing variability that may affect learning performance (36, 63).

The number of conditioning loops reported in the literature varies from one to 12 (60); in our study, five loops were used. Finally, the pressure of B2 enabled the memory retention tests to begin. In this step the sole CS was delivered to the restrained honey bee for 6 s. Pressing button B1 within 2 s recorded a positive proboscis extension.

Circuit diagrams (**Supplementary Figure S2**) were designed using Fritzing software (http://fritzing.org) and edited in CorelDRAW X3. The Arduino Integrated Development Environment (IDE) software based on Processing IDE (http://processing.org) was used to programme the device (70). An USB cable connected the board to a laptop. The software PuTTY (release 0.76; (60)) was used to display the data on the monitor and, at the same time, to save the data log to a text file. A value of ‘1’ was automatically assigned when B1 was pressed within the required time window and meant a positive response to olfactory PER conditioning or memory retention test. In contrast, “0” was automatically assigned in case there was no pressure on the button and meant no response of the honey bee.

## 3 Results

**Figure 2:**
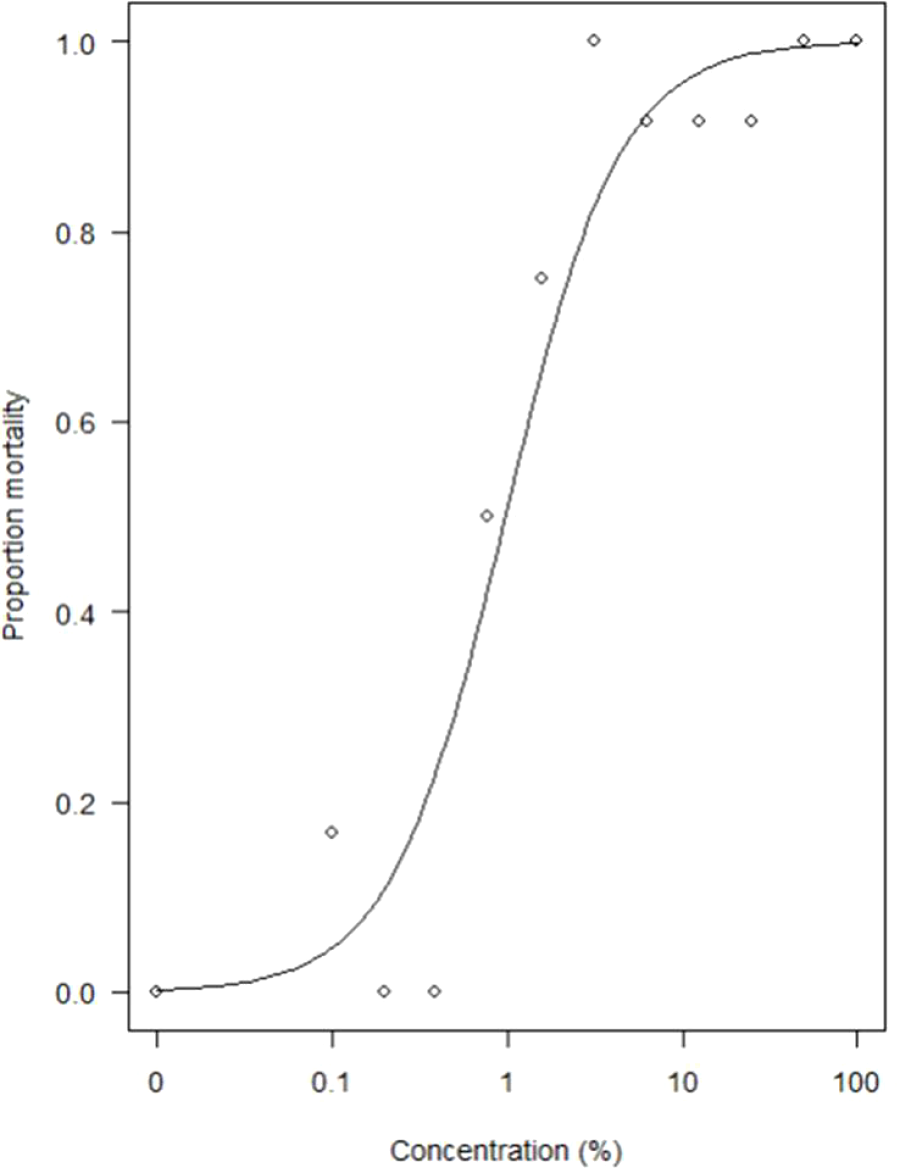
Dose response curve of honey bee mortality after exposure to increasing concentration of spinosad, with 100% of the field dose of the commercial product used.

Dose-response mortality analysis revealed an increased mortality of honey bees at increased concentration of the spinosad-based bioinsecticide (**Figure 2**). From the fitted dose–response curve, the estimated concentrations causing mortality of 0.2% and 20% corresponded to 0.023 and 0.43% of the field dose, respectively.

PER response during the conditioning trials was similar for honey bees exposed to LC_0.2_ (37.0% ± 1.92) and LC_20_ (41.3% ± 2.13), which were lower compared to control (82.0% ± 1.92) (results of GLMs followed by multiple comparisons procedure in **Table 1**).

**Table 1:** Positive response (%) of honey bees treated with different concentrations (LC) of spinosad and evaluated at different conditioning trials.

LC	trial.1	trial.2	trial.3	trial.4	trial.5
0	80.0 a	88.3 a	81.7 a	83.3 a	76.7 a
0.2	31.7 b	38.3 b	41.7 b	33.3 b	40.0 b
20	41.7 b	41.7 b	48.3 b	35.0 b	40.0 b

Percentage values with different letters are significantly different for P ≤ 0.05 according to GLM (binomial distribution) followed by multiple comparisons procedure.

**Figure 3:**
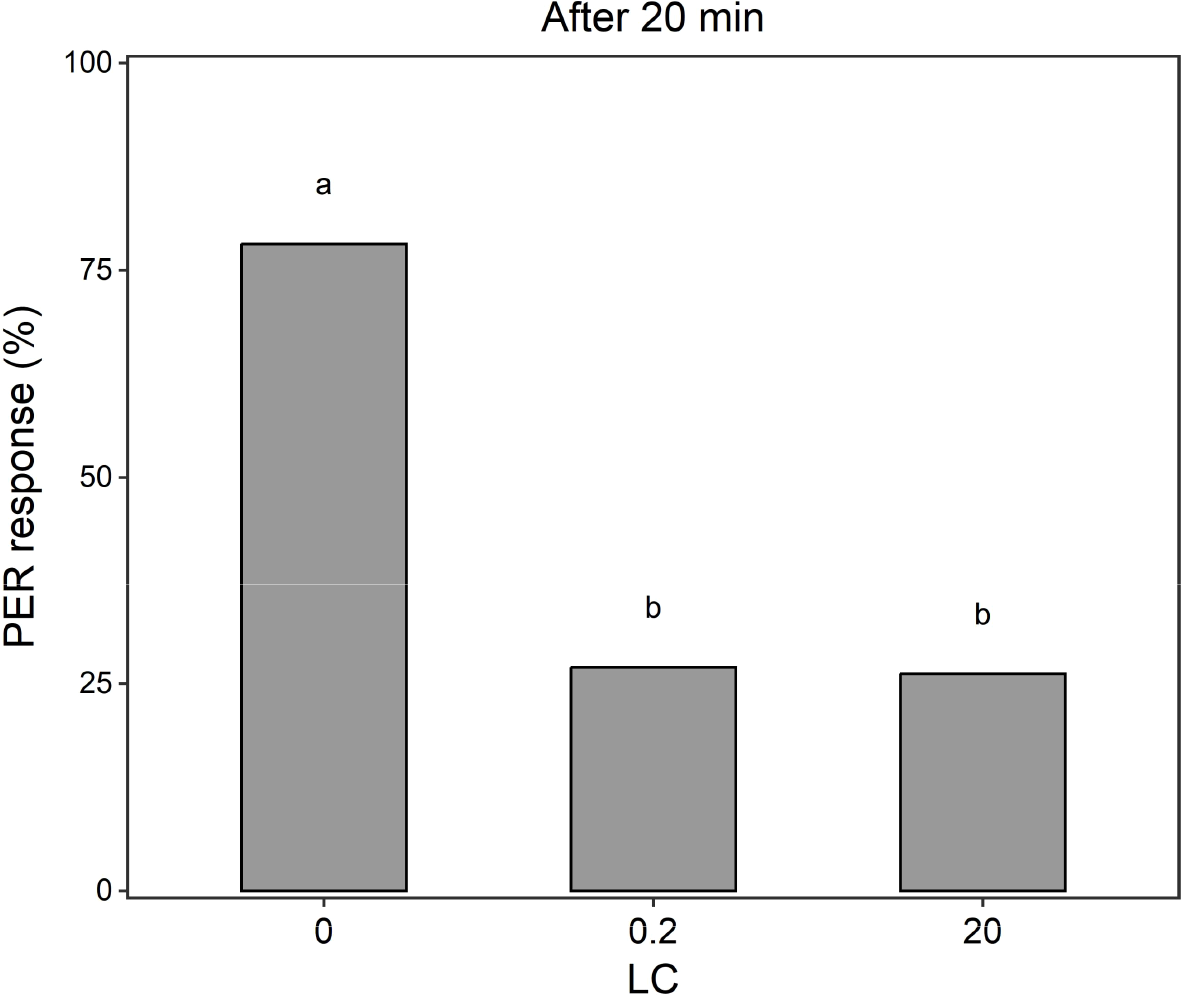
Bar chart of the memory retention test results in honey bees, 20 min after the last conditioning trial. Positive response (%) of honey bees treated with different concentrations (LC) of spinosad. Percentage values with different letters are significantly different for P ≤ 0.05 according to GLM (binomial distribution) followed by multiple comparisons procedure.

In the memory retention test, treatment with spinosad-based insecticide affected PER at 20 min after the last conditioning trial (binomial GLM, χ² = 36.31, P < 0.001). Honey bees exposed to LC_0.2_ and LC_20_ exhibited similar PER responses, which were lower compared to the control (LC_0_) (results of GLMs followed by multiple comparisons procedure in **Figure 3**).

## 4 Discussion

The reduced PER responses at 20 min after the last trial suggest that spinosad impairs memory in honey bees. The low PER response at LC_0.2_ suggests that even very low exposure disrupts memory. This supports the hypothesis that cognitive functions are sensitive to sublethal spinosad exposure and also that impairment may occur independently of concentration, within the range of concentrations tested. Our findings add a further dimension to the known detrimental effects of this natural insecticide on honey bee survival, food intake, and behaviour under laboratory conditions (10, 11, 71, 72).

Araújo et al. (72) showed that oral exposure to spinosad reduced survival, food consumption, flight behaviour and respiration rate, while altering oxidative and immune-related parameters in forager bees. On the other hand, under field conditions Miles (73) did not observe any significant effect on the survival of honey bees exposed to spinosad residues. Morandin et al. (74) noticed that *Bombus impatiens* Cresson adults exposed to this natural insecticide during larval development exhibited slower foraging on complex floral resources. Similar evidence was detected in the stingless bee *Plebeia lucii* Moure, in which spinosad exposure reduced walking and flight activity (75). Taken together, these studies indicate that sublethal exposure to spinosad can disrupt movement as well as foraging behaviour.

In addition, Carvalho et al. (76), after acute contact exposure of *A. mellifera* workers to 2.36 and 4.71 ng bee⁻¹ of spinosad, reported a modulation of enzyme biomarkers such as CaE-1, CaE-2, GST, CAT and ALP, indicating the activation of a physiological stress response. Our results also revealed a dose-response relationship, with increasing concentrations of spinosad resulting in higher mortality rate of *A. mellifera*.

The high acute toxicity observed, with a DL_50_ close to 1% of the field dose, is consistent with previous evidence indicating that *A. mellifera* is highly susceptible to spinosad under direct exposure conditions. Indeed, published data report strong acute toxicity by both contact and ingestion (e.g., 24 h oral LC_50_ of 7.34 mg AI L^−1^), whereas field studies suggest that actual mortality may be lower when bees are exposed only to dry rather than fresh residues (reviewed by 10).

Neurotoxic insecticides can alter hymenopteran ability to recognise, learn, and memorise host-associated chemosensory molecules (77–80). The effects of synthetic insecticides on learning behaviour and memory retention have been studied in bees (11, 81–83) and hymenopteran parasitoids (58, 84–86). A sublethal dose of sulfoxaflor, a compound belonging to sulfoximine insecticides, can adversely affect honey bee learning and memory when applied before or after conditioning (39). Impaired memory was also demonstrated in *A. mellifera* and *A. mellifera jemenitica* Ruttner foragers that were fed a sucrose solution containing a sublethal dose of the neonicotinoid imidacloprid (57, 87). High concentrations of flupyradifurone damage the learning and memory abilities of honey bee pollen or nectar foragers (88). Although considered safer alternatives to synthetic insecticides, evidence from several papers indicates that also natural pesticides are not inherently without risk for honey bees (7, 89, 90). For instance, caution should be paid in the use of oregano oil, an essential oil used for insect pest management, as it reduces the survival of *A. mellifera* (91, 92). Overall, Cappa et al. (12) and Giunti et al. (13) reviewed the effects of several biopesticides on *A. mellifera*, highlighting mortality or detrimental effects on survival rates. Carlesso et al. (44) showed that, although *B. bassiana* did not directly affect memory, less fungus-exposed honey bees than bees in the control group responded to odorants, possibly as a result of deficiency in odour coding.

An additional result of our investigation is the development and evaluation of a microcontroller-based device that can be used to assess learning behaviour in honey bees in a rapid and reproducible manner, while minimising handling by the operator. To our knowledge, this is the first report demonstrating that this device can effectively assess the consequences of sublethal insecticide exposure on olfactory conditioning and memory of as many as 20 honey bees at a time. Our automated system allowed an accurate application of the PER protocol with a fine control of CS-US overlap resulting in consistent results. Shepherd (43) provided a valuable early example of a turntable-based approach for PER conditioning of 12 bees within the same experimental cycle. We extended this concept by introducing microcontroller-driven rotation, automated airflow delivery for 20 honey bees. Bragunde et al. (48) presented an open-source microcontroller-based system for automated odour delivery during PER conditioning of individual honey bees. However, our device integrates a motorised turntable enabling the sequential conditioning of 20 bees per cycle, crystal-oscillator-controlled timing, and real-time digital data recording via serial transfer. Among existing devices, the system by Strelevitz et al. (47) offers the most complete automation, including AI-based recognition of PER outcomes. However, their setup relies on a pre-trained convolutional neural network, which is only partially publicly available, and on MATLAB, a proprietary commercial software, which together limit the replicability of the system and the possibility for end-user customisation. In contrast, our device is based entirely on free, open-source tools (Processing IDE and PuTTY), ensuring full replicability and flexibility for adaptation by other laboratories without specialised computational expertise or costly software licences. The proboscis extension reflex behaviour is widely exploited for evaluating learning and memory retention in honey bees. However, the inter-stimulus interval i.e. the time between the CS (linalool in this study) delivery and the US (sucrose solution), represents a pivotal step that can influence the learning and memory performance (34, 36, 63, 93).

The device can be used for the rapid collection of behavioural data aiming at assessing crucial questions in ecology and ecotoxicology. A larger number of biological pesticides are available on the market because of the ban of some synthetic molecules (1). Adopting the present protocol can help in gaining new data that would likely support policy makers to better understand possible effects of new pesticides on honey bees (94).

Honey bees are also capable of visual learning and can be trained by exposing them to different light colours (28, 95, 96). Light sources (LEDs) can be easily integrated into the device, which, after reprogramming, would allow the contemporary assessment of olfactory and visual stimuli in the honey bee learning process. Another interesting application envisages learning behaviour in pollinators other than honey bees. With small modifications, different plastic tubes can be used to support large-size pollinators inside cages, allowing for the observation of multiple PER behaviours simultaneously in, for example, bumblebees or solitary bees, for which data are relatively scarce (1, 97, 98). Further adaptations could be considered for other insect groups, for example, Lepidoptera and Diptera, that have been recognised as good models for studying PER response (99, 100). The device can also support a rigorous assessment of scientific hypotheses in fundamental research, such as the presence of lateralisation (different response of left vs. right antenna) or gene expression analysis (69, 101–103). Nonetheless, screening 20 insects at a time is likely to enhance standardisation and intra-cycle replication. The observation of 20 honey bees within the same experimental cycle represents a significant improvement on previous Arduino-based automated devices (47, 48). However, there are some limitations and ameliorations to the setting that could be implemented. In this current configuration, the device is equipped with an air tank and needs 220V electricity, hampering its easy use in the wild. However, the air tank and the solenoid valve could be replaced with environmental air, which can be inflated by a low voltage diaphragm pump and purified by an activated charcoal filter cartridge. The 220V could be replaced with 6V batteries that can be independently plugged to the board, the servo, and the stepper motor. Although the use of a preassembled board facilitated the setup of the system, a minimal configuration with only the ATmega 328p and the bare essential can also be considered to minimise the power consumption if the device is to be powered by batteries. Ideally, device customisation for field usage would represent a great advantage because it would allow for a rapid evaluation of pollinator behaviour directly in their natural environment. Concerning the evaluation of the sublethal effects of spinosad, here we focussed on the short-term memory (20 min time point) (87, 104), rather than late long-term memory (105, 106), which should be evaluated in future studies. Assessing potential unintended pesticide contamination in each honey bee group before the bioassays would help disentangle the effects of pesticide residues from those of the artificial treatments. Future studies should consider potential re-conditioning trials of the same harnessed bee by using non-reinforced probe tests or independent cohorts for each retention interval to provide fully comparable estimates of memory decay over time.

In conclusion, our device represents a powerful and reliable tool for investigating the possible harmful effects of chemicals on beneficial insects. By providing time-controlled exposure to stimuli and the subsequent accurate observation of PER behaviour, the device can allow the detection of sublethal effects of pesticides that can adversely affect the behaviour of beneficial insects. Ultimately, it can potentially be used in risk assessment process, contributing to the design of sustainable and safe pest management strategies.

## Data Availability

The raw data supporting the conclusions of this article will be made available by the authors, without undue reservation.
