# Supplementary material for: Assessing spinosad effect on honey bee olfactory conditioning using a microcontroller-based device
Source: Front Insect Sci. 2026 May 14;6:1785989. doi: 10.3389/finsc.2026.1785989 (PMC13215915; doi:10.3389/finsc.2026.1785989)
Supplement: Supplementary file 1 [file SupplementaryFile1.docx]

Supplementary Material

Table S1. List of components used for the device.

| Component |
| --- |
| Unipolar stepper motor, Vexta PK245-2B |
| Servo motor DS3225 |
| Electrovalve (12V) |
| Magneto-thermic switch (16A) |
| AC/DC power supply (4.5 – 5.5 V) |
| AC/DC power supply (11.5 – 13.5 V) |
| ATmega 328p embedded in Arduino Uno (Rev. 3) board |
| Motor controller L293D |
| Relay (5VDC) |
| Capacitor (47 uF) |
| Resistors (1 × 1 kΩ; 2 × 10 kΩ) |
| Diod |
| LED |
| Push buttons (2) |
| Electrical wires |
| USB cable |
| Medical grade air tank and pressure gauze |
| Flowmeters (2 × 0-2 L/min range) |
| Dreschel bottles (2 × 250 mL) |
| Silicone tubes |
| Glass or plastic Y-shape connectors / glass pipe |
| Plastic turntable, black boxes, plastic tubes, and pins for restraining bees |
| Metal grid, black cardboard, fluorescent lights |

**Figure S1.** Overview of the device. “ai” = pressurised medical grade air tank; “pg” = pressure gauge; “v” = solenoid valve; “fl1” and “fl2” = different regulated flowmeters; “hu”=humidifier; “st” = Y-shaped glass connector containing filter paper soacked with the conditioned stimulus; “s” = servo motor controlling the glass pipe that delivers the airflow to the antennae of the honey bee; “m” = stepper-motor-rotated turntable with 20 small cages affixed, each containing a restrained honey bee; “co” = command control with two buttons and a LED light; “eb” = electrical box; “usb” = USB connector used to send out real time PER response data to a pc; “ea” = an electric aspirator to prevent odour saturation. A metal grid cage was added around the turntable and was surrounded by a black cardboard to minimise visual input from the room (not shown). Video available at: <https://spaces.hightail.com/space/G3x6odCrSz>


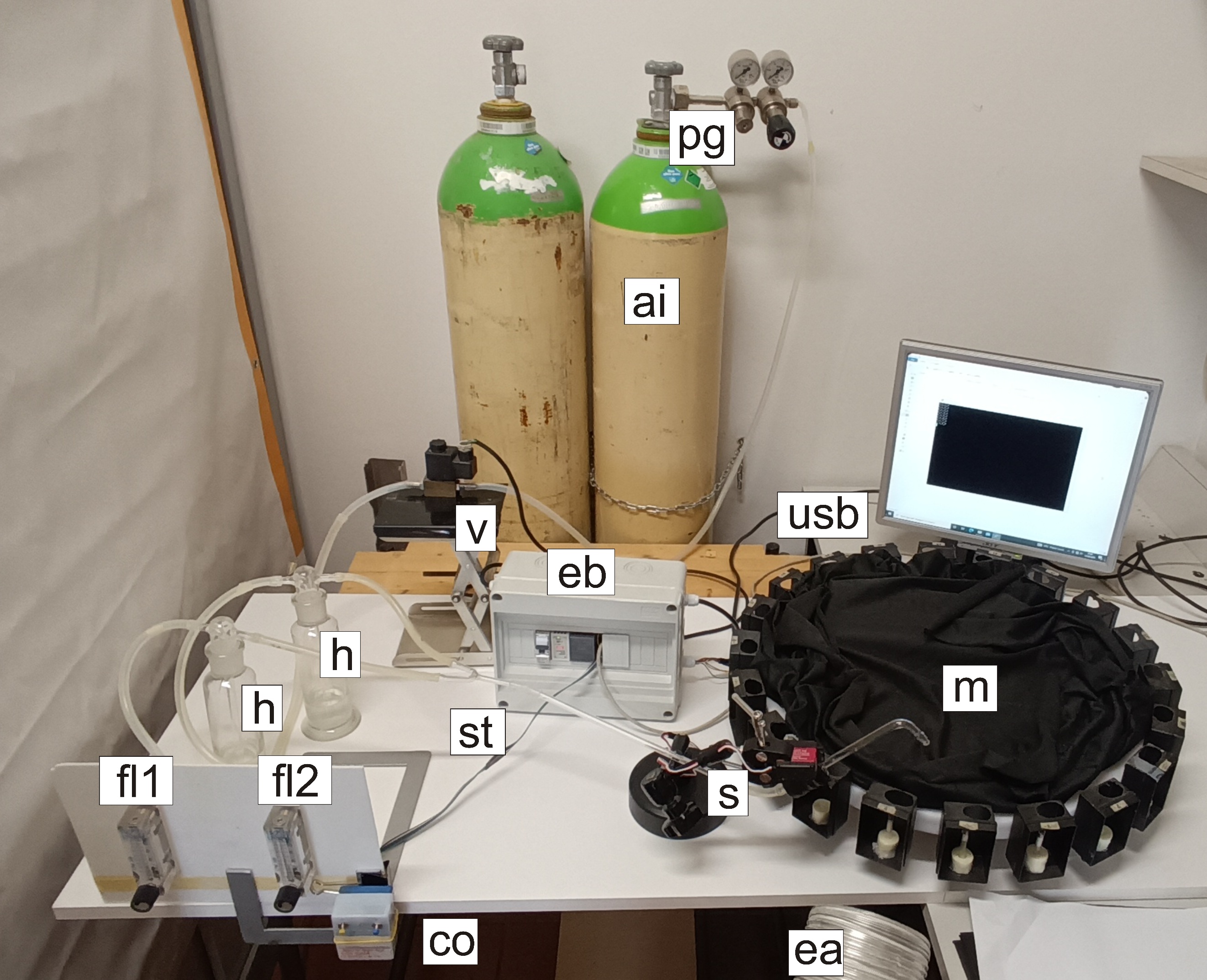


**Figure S2.** Circuit diagram of the designed automatic system.

**
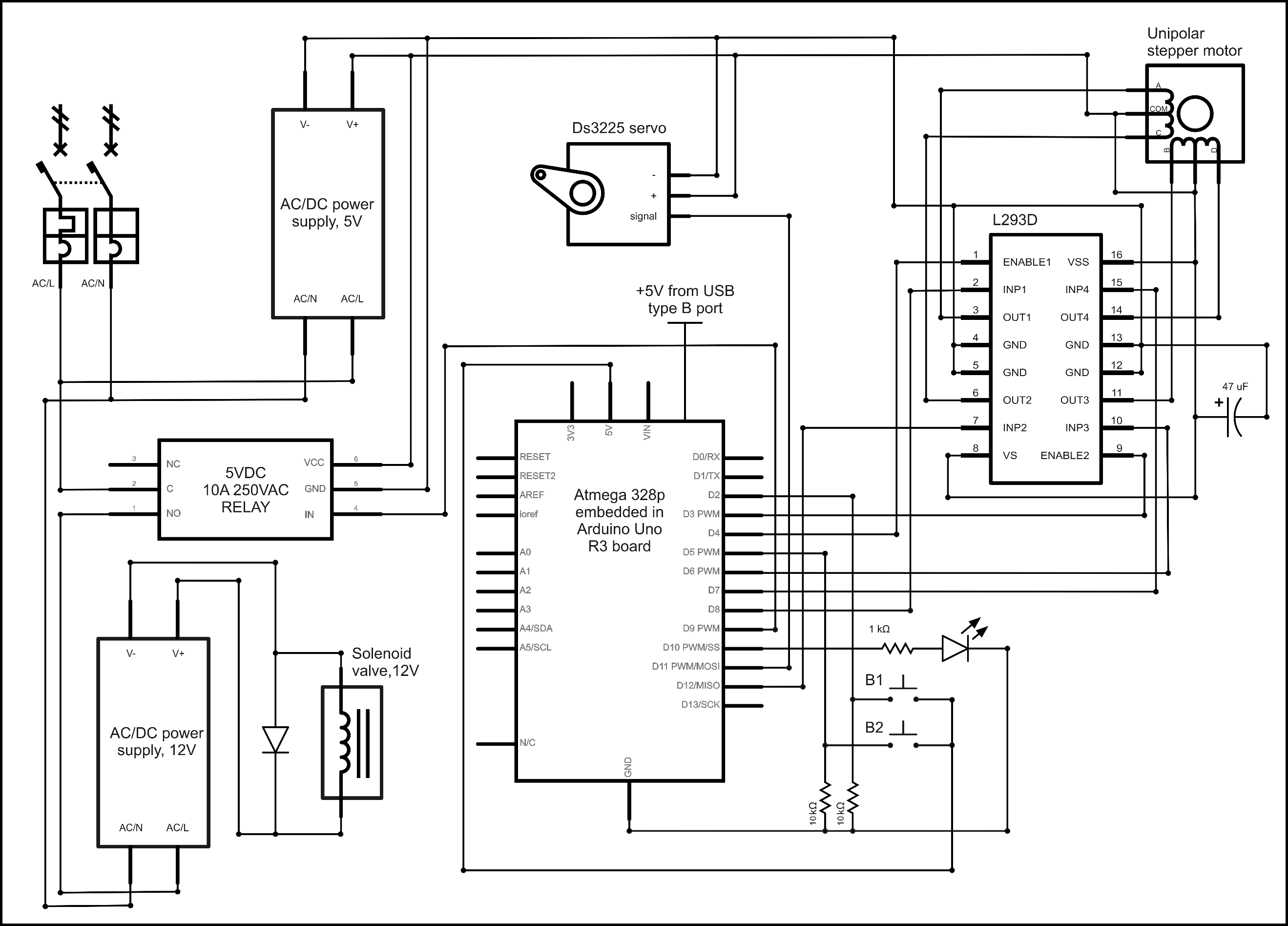
**

Programming code

/*

This sketch controls the rotative part of the device used for evaluating PER response of honey bees.

The rotative part has 20 slots, each for an individual honey bee.

*/

#include <Servo.h>

#include <Stepper.h>

Servo myservo;

#define STEPS 200

// VEXTA unipolar motor has 200 steps, 1.8 degrees per step.

// Initialise the stepper library

stepper stepper (STEPS, 8, 11, 6, 7 );

const int enable12 = 3; //bridge 1 and 2 enable (pin 1 on L293D)

const int enable34 = 4; //bridge 3 and 4 enable (pin 9 on L293D)

const int ButtonPin1 = 2; // the pin to which the push button is attached

const int ButtonPin2 = 5; // the pin to which the push button is attached

const int LED1 = 10; // the led that switches on when the antennae are touched with a fine brush

const int RELE = 11; // the relay that activates the air pump for 15 sec or 6 sec of airflow delivery

int ButtonState1 = 0; // current state of the button

int lastButtonState1 = 0; // previous state of the button

int ButtonState2 = 0; // current state of the button

int lastButtonState2 = 0; // previous state of the button

int lno = 0; // current state of button1

int lastlno = 0; // previous state of button1

int lyes = 0; // current state of button2

int lastlyes = 0; // previous state of button2

int ccond =0; // number of conditionings

int servo_pin = 9;

int pos = 10;

int myArray[20]={30, 30, 30, 30, 30, 30, 30, 30,30, 30, 30, 30, 30, 30, 30, 30,30,30,30,30}; //modify calibrated position according to irregularities of the turntable

void setup() {

// initialise the serial port:

Serial.begin(9600);

stepper.setSpeed(1);// (n) means: (1/n)* 60s is the time the turntable spends to complete a 360° rotation

pinMode(enable12, OUTPUT); //Set the enable12 pin to Output

pinMode(enable34, OUTPUT); //Set the enable34 pin to Output

pinMode(ButtonPin1, INPUT); //Set the button1 to Input

pinMode(ButtonPin2, INPUT); //Set the button2 to Input

pinMode(LED1, OUTPUT); //Set the LED1 to Output

pinMode(RELE, OUTPUT); //Set the RELE to Output

myservo.attach(servo_pin);

myservo.write(0);

}

void loop() {

digitalWrite(enable12, HIGH);

digitalWrite(enable34, HIGH);

digitalWrite(RELE, LOW);

ButtonState1 = digitalRead(ButtonPin1);

ButtonState2 = digitalRead(ButtonPin2);

if(ButtonState1!=lastButtonState1) {

stepper.step(0.1*STEPS); // at the beginning, allow 20 steps rotation and verify the

//correct alignment of the glass tube with the top opening of the black cage

delay(5000);

for(pos = 10; pos < 29; pos += 1){myservo.write(pos); delay(50);}

delay(20000);

for(pos = 29; pos>10.5;pos-=1){myservo.write(pos); delay(50);}

delay(2000);

for (int x = 0; x < 20; x++){

stepper.step(0.05*STEPS);

delay(2000);

for(pos = 10; pos < 29; pos += 1){ myservo.write(pos); delay(50);}

delay(5000);

digitalWrite(RELE, HIGH);

delay(15000);

digitalWrite(RELE, LOW);

delay(2000);

lyes = digitalRead(ButtonPin1);

if(lno<lyes) {

digitalWrite(LED1, HIGH); delay(50); digitalWrite(LED1, LOW);delay(100);digitalWrite(LED1, HIGH);delay(50);digitalWrite(LED1, LOW);delay(100);digitalWrite(LED1, HIGH);delay(50);digitalWrite(LED1, LOW);}

for(pos = 29; pos>10.5;pos-=1){myservo.write(pos); delay(50);}

//x-1 because the first array position is 0->0-19

Serial.print("a_");

Serial.print("0_");

Serial.print(x+1);

Serial.print("_");

Serial.print(1-lyes); //0 means to remove it; 1 means to keep it

Serial.println();

delay(2000);

}

}

if(ButtonState2!=lastButtonState2) {

ccond = ccond+1;

for (int y = 0; y < 20; y++){

stepper.step(0.05*STEPS);

delay(2000);

for(pos = 10; pos < 29; pos += 1){ myservo.write(pos); delay(50);}

delay(5000);

digitalWrite(RELE, HIGH);

delay(3000);

digitalWrite(LED1, HIGH);

delay(3000);

digitalWrite(LED1, LOW);

digitalWrite(RELE, LOW);

delay(2000);

lyes = digitalRead(ButtonPin1);

if(lno<lyes) {

digitalWrite(LED1, HIGH); delay(50); digitalWrite(LED1, LOW);delay(100);digitalWrite(LED1, HIGH);delay(50);digitalWrite(LED1, LOW);delay(100);digitalWrite(LED1, HIGH);delay(50);digitalWrite(LED1, LOW);}

for(pos = 29; pos>10.5;pos-=1){myservo.write(pos); delay(50);}

//x-1 because the first array position is 0->0-19

Serial.print("c_");

Serial.print(ccond);

Serial.print("_");

Serial.print(y+1);

Serial.print("_");

Serial.print(lyes);

Serial.println();

for(pos = 29; pos>10.5;pos-=1){myservo.write(pos); delay(50);}

delay(2000);

}

}

ButtonState1=lastButtonState1;

ButtonState2=lastButtonState2;
